# Appendicitis with submucosal fecalith mimicking a submucosal tumor: a case report

**DOI:** 10.1186/s40792-021-01169-9

**Published:** 2021-04-27

**Authors:** Tomoaki Bekki, Toshikatsu Fukuda, Toshiyuki Moriuchi, Yosuke Namba, Sho Okimoto, Shoichiro Mukai, Yasufumi Saito, Koichi Oishi, Toshihiro Nishida, Hideki Ohdan

**Affiliations:** 1 Department of Surgery, Chugoku Rosai Hospital, Hiroshima, Japan; 2 Department of Pathology, Chugoku Rosai Hospital, Hiroshima, Japan; 3 Department of Gastroenterological and Transplant Surgery, Applied Life Sciences, Institute of Biomedical and Health Sciences, Hiroshima University, Hiroshima, Japan

**Keywords:** Appendicitis, Submucosal fecalith, Interval appendectomy

## Abstract

**Background:**

Submucosal fecalith(s) mimicking submucosal tumors of the gastrointestinal lumen are rare. Moreover, accurate preoperative diagnosis of these entities is exceedingly difficult, and the mechanism(s) of their formation remains unclear.

**Case presentation:**

A 40-year-old woman visited the authors’ hospital due to lower abdominal pain and diarrhea. She had previously been treated for endometriosis. Laboratory investigation revealed increased C-reactive protein levels. Abdominal contrast-enhanced computed tomography revealed thickening of the appendix wall and dilation of the small intestine. The patient was diagnosed with paralytic ileus caused by appendicitis, and interval appendectomy was scheduled. She underwent laparoscopic-assisted appendectomy after conservative treatment. Partial cecal resection was selected due to the presence of an elastic mass in the cecum. The final pathological diagnosis was submucosal fecalith, not submucosal tumor. On day 5, the patient was discharged without any postoperative complications.

**Conclusions:**

In cases of appendicitis with fecalith(s) that appear as submucosal tumor(s) on colonoscopy, submucosal fecalith mimicking submucosal tumor should be considered in the differential diagnosis.

## Background

Appendicitis with fecalith(s) is often encountered in clinical practice. The ratio of appendicitis cases with fecalith has been reported in different studies [1–4], some of which have reported an association between the appendix and fecalith formation, and that fecalith formation is associated with perforation in cases of acute appendicitis and the failure of conservative treatment [5–7]. Others have demonstrated that fecaliths do not influence gangrenous or perforated appendicitis [3]. Nevertheless, the influence of fecalith(s) on the occurrence of appendicitis remains controversial.

Interestingly, sometimes intraluminal fecaliths show an extremely rare form. Only a few case reports [8–18] have described fecaliths mimicking submucosal tumor(s). The present report describes a case of appendicitis with submucosal fecalith mimicking a submucosal tumor.

## Case presentation

**Fig. 1:**
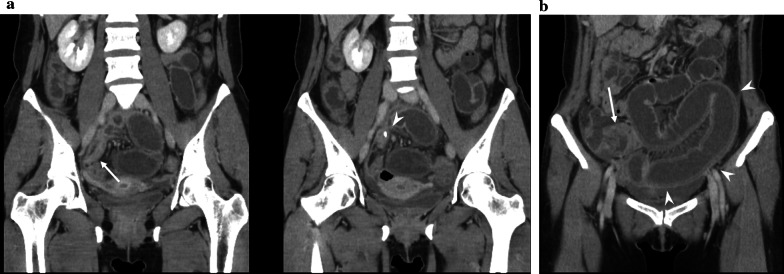
Abdominal contrast-enhanced computed tomography findings. **a** An appendix wall (white arrow) was thickened and there was a fecalith (white arrowhead) at the appendix root. **b** The cecum (white arrow) was edematous, and the small intestine in the pelvis (white arrowhead) was dilated

**Fig. 2:**
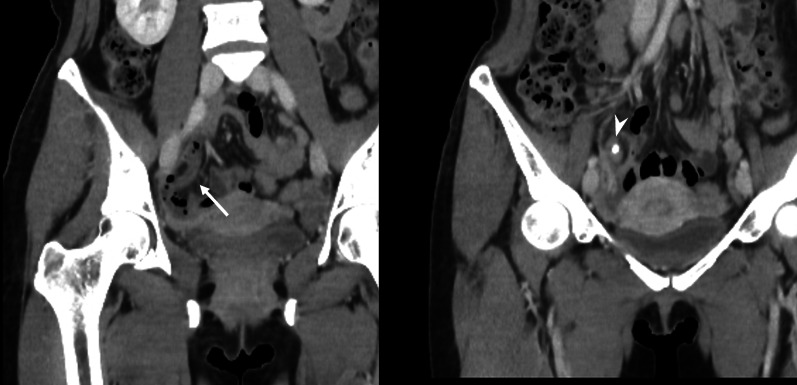
Abdominal contrast-enhanced computed tomography findings after conservative treatment. The wall thickness and swelling of the appendix (white arrow) were improved. The fecalith (white arrowhead) remained near the root of the appendix

**Fig. 3:**
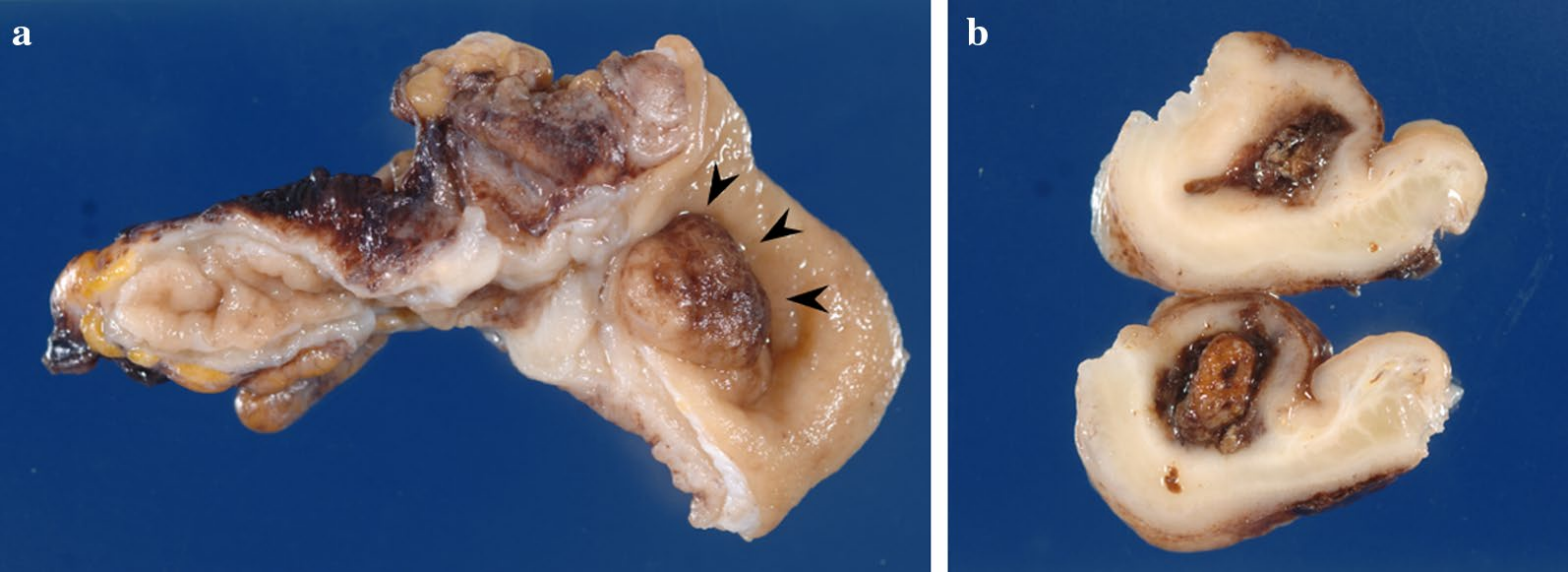
Macroscopic findings. **a** A mass similar to the submucosal tumor (black arrowhead) was detected near the appendix root. **b** A fecalith measuring 12 mm was detected in the submucosal layer

**Fig. 4:**
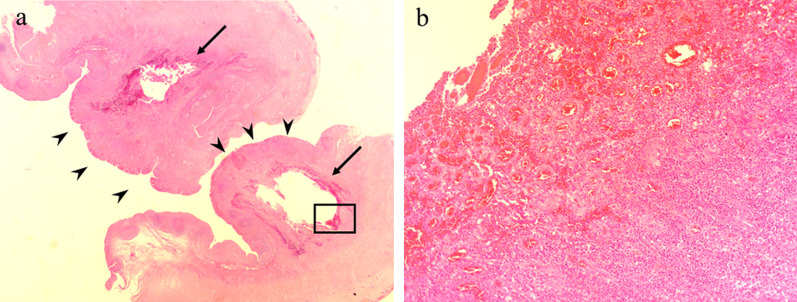
Histopathological findings. **a** The white spaces (black arrow) are the positions where the fecalith existed. There was a mucosa (black arrowhead) only above the fecalith. (Hematoxylin–eosin stain, original magnification × 40.) **b** An enlarged image with a square on a. No epithelial cells were observed. Hematoma components and numerous inflammatory cells were observed. (Hematoxylin–eosin stain, original magnification × 200)

A 40-year-old woman was admitted to the department of surgery at the authors’ hospital with complaints of lower abdominal pain and diarrhea. She also exhibited fever and abdominal distension. She had no relevant surgical history and was being treated for endometriosis with low-dose estrogen progestin (LEP). Although her white blood cell count was normal, C-reactive protein (CRP) levels were extremely elevated (20.36 mg/dL). Abdominal ultrasonography revealed dilationand extensive fluid retention in the small intestine. Contrast-enhanced abdominal computed tomography (CT) revealed appendiceal wall thickening with fecalith (Fig. 1a), edema in the cecum, dilation of the small intestine in the pelvis (Fig. 1b), and ascites around the liver. There was no abscess in the pelvis or constriction of the bowel. Vaginal cultures for gonorrhea and chlamydia were negative. Initial diagnosis was suspicious for paralytic ileus caused by pelvic peritonitis due to appendicitis rather than gynecological infection. Conservative treatment with antibiotics and an ileus tube were selected for two reasons. First, the probability of ileocecal resection was high due to cecum edema. Second, she was at high risk for thrombosis due to the use of LEP for endometriosis. Conservative treatment was highly effective. On day 9 of admission, CRP levels dramatically improved (0.73 mg/dL). The patient resumed a regular diet 5 days after the start of treatment and, thereafter, her clinical course was uneventful, and she was discharged 12 days after admission. Five days after discharge, she was re-examined and laboratory investigations performed at the outpatient clinic revealed normal CRP levels (0.04 mg/dL). On contrast-enhanced abdominal CT, which was performed 2 weeks after discharge, swelling in the appendix was improved, although the fecalith remained near the appendix root (Fig. 2). There were no CT findings suggestive of malignancy. She underwent laparoscopic-assisted interval appendectomy without preoperative colonoscopy 2 months after starting treatment. The operative findings revealed no adhesion between the appendix and surrounding organs. An elastic mass was confirmed in the cecum near the root of the appendix. Accordingly, partial cecal resection was performed to excise the mass. The total duration of the operation was 73 min, and intraoperative blood loss was minimal. Macroscopically, a mass similar to a submucosal tumor was found in the cecum near the appendix root (Fig. 3a). The fecalith, measuring 12 mm in size, was detected in the submucosal layer (Fig. 3b). No appendiceal swelling was observed. Histopathologically, inflammation had spread around the fecalith, with no malignancy (Fig. 4a, b). The postoperative course was uneventful, and the patient was discharged on postoperative day 5.

## Discussion

There are multiple types of submucosal lesions, including lipomas, lymphomas, gastrointestinal stromal tumors, and appendiceal tumors [19]. The histological diagnosis of submucosal lesions is difficult with colonoscopy and endoscopic biopsy. In addition, Kangaspunta et al. demonstrated that preoperative abdominal CT was poor in detecting tumors in patients with acute appendicitis[20]. Since 1981, several studies have described submucosal fecalith(s) mimicking submucosal tumors, as shown in Table 1 [8–18]. Some characteristics of the 12 patients with submucosal fecalith included in our brief literature review included a mean age of 47 years (range, 6–74 years), a female-to-male prevalence ratio of 1:2, and the fecalith was located around the cecum. However, to the best of our knowledge, there have been no reports of preoperative diagnosis of submucosal fecalith.

**Table 1:** Review of diagnosed cases of submucosal fecalith

Case	Year of publication	Author	Patient age	Sex	Fecalith position	Treatment	Preoperative diagnosis	Clinical diagnosis	Diagnostic modality
1	1981	Gohar [8]	6	F	Cecum	Operation	–	Intramural or submucosal mass	Radiography, barium enema
2	1987	Ito [9]	57	F	Cecum	Operation	–	Malignant submucosal tumor	Radiography, barium enema, CS, biopsy
3	1995	Kimura [10]	73	M	Cecum	Operation	–	Malignant submucosal tumor	Radiography, CS, barium enema, EUS, biopsy
4	2009	Lee [11]	44	M	Appendiceal orifice	Endoscopic resection	–	Submucosal tumor	CS, ACT, EUS, biopsy
5	2013	Alhalabi [12]	41	M	Cecum	Operation	–	Submucosal mass	CS, ACT, EUS, biopsy
6	2014	Meguro [13]	73	M	Appendiceal orifice	Operation	–	Appendiceal intussusception	CS, ACT, biopsy
7	2014	Zhao [14]	30	M	Ileocecal valve	Endoscopic resection	–	Submucosal mass	ACT, CS, EUS,
8	2017	Ruan [15]	65	F	Cecum	Operation	–	Appendicitis with fecalith	AUS, ACT,
9	2018	Kramaer [16]	26	M	Cecum	Operation	–	Appendicolith	CS, ACT, biopsy
10	2018	Narashima [17]	74	M	Appendiceal orifice	Operation	–	Carcinoid tumor, mucocele, gist	CS, ACT, biopsy
11	2019	Bustamante [18]	34	M	Cecum	Operation	–	Malignant submucosal mass	CS, ACT, biopsy
12	2020	Our case	40	F	Appendiceal orifice	Operation	–	Appendicitis with fecalith	ACT

Appendiceal cancers are rare, with previous studies reporting that they account for < 1% of all gastrointestinal cancers [21, 22]. Some factors have been reported to contribute to the risk for appendiceal neoplasm and cancer, including age > 40 years, appendiceal diameter > 10 mm, and complicated appendicitis [23, 24]. In addition, some studies have demonstrated that interval appendectomy increases the risk for appendiceal neoplasm and malignancy [25, 26]. Although the pathological diagnosis in our case was not malignancy, preoperative colonoscopy should have been performed due to the age of the patient (i.e., approaching middle age). We recommended preoperative colonoscopy, but this was refused by the patient. Though this was a benign lesion, the patient should have undergone preoperative colonoscopy to exclude malignant lesions due to age factors, even if there were no malignancy findings on CT.

Although various mechanisms have been proposed, the exact mechanisms of submucosal fecalith formation remain unclear. Alhalabi et al. suggested that fecalith(s) could be trapped in the appendiceal orifice and develop in the submucosal layer over time [12]. Bustamante et al. suggested that the result of long-term incarceration of an appendicular fecalith or the constant accumulation of feces through fissure(s) in the mucosa cause submucosal fecalith [15, 18]. Ito et al. favored the hypothesis that intussusception of the appendix, which excludes foreign bodies from the lumen of the appendix, may lead to submucosal fecalith. Nevertheless, the exact mechanism of submucosal fecalith formation remains controversial. In our case, there was no mucosa except the upper fecalith; therefore, it was difficult, to presume that the fecalith was trapped in the cecal diverticulum. In addition, it was difficult to presume obstruction of the appendix root due to submucosal fecalith in the cecum because there were no findings of appendiceal swelling. Based on intraoperative findings, the invaginated appendix was not the cause of the submucosal fecalith. The mechanism in our case was inferred from the clinical course and histopathological findings, which suggested high-grade inflammation of the appendix root due to fecalith incarceration. Fecalith incarceration in the appendix root induces high levels of inflammation with ulceration, which in turn causes pelvic peritonitis. During tissue repair, the surface of the fecalith was covered with epithelium, which appeared as a submucosal tumor.

## Conclusions

We encountered a rare case of submucosal fecalith mimicking a submucosal tumor. In cases of appendicitis with fecalith—in which colonoscopy findings suggest submucosal tumor—it is important that the chosen surgical strategy is not overly invasive.

## Data Availability

No applicable.

## Abbreviations

LEP: Low-dose estrogen progestin
CRP: C-reactive protein
CT: Computed tomography
